# Detecting inpatient falls by using natural language processing of electronic medical records

**DOI:** 10.1186/1472-6963-12-448

**Published:** 2012-12-05

**Authors:** Shin-ichi Toyabe

**Affiliations:** 1 Niigata University Crisis Management Office, Niigata University Hospital, Asahimachi-dori 1-754, Chuo-ku, Niigata City, 951-8520, Japan

**Keywords:** Natural language processing, Text mining, Falls, Adverse events, Incident reports

## Abstract

**Background:**

Incident reporting is the most common method for detecting adverse events in a hospital. However, under-reporting or non-reporting and delay in submission of reports are problems that prevent early detection of serious adverse events. The aim of this study was to determine whether it is possible to promptly detect serious injuries after inpatient falls by using a natural language processing method and to determine which data source is the most suitable for this purpose.

**Methods:**

We tried to detect adverse events from narrative text data of electronic medical records by using a natural language processing method. We made syntactic category decision rules to detect inpatient falls from text data in electronic medical records. We compared how often the true fall events were recorded in various sources of data including progress notes, discharge summaries, image order entries and incident reports. We applied the rules to these data sources and compared F-measures to detect falls between these data sources with reference to the results of a manual chart review. The lag time between event occurrence and data submission and the degree of injury were compared.

**Results:**

We made 170 syntactic rules to detect inpatient falls by using a natural language processing method. Information on true fall events was most frequently recorded in progress notes (100%), incident reports (65.0%) and image order entries (12.5%). However, F-measure to detect falls using the rules was poor when using progress notes (0.12) and discharge summaries (0.24) compared with that when using incident reports (1.00) and image order entries (0.91). Since the results suggested that incident reports and image order entries were possible data sources for prompt detection of serious falls, we focused on a comparison of falls found by incident reports and image order entries. Injury caused by falls found by image order entries was significantly more severe than falls detected by incident reports (p<0.001), and the lag time between falls and submission of data to the hospital information system was significantly shorter in image order entries than in incident reports (p<0.001).

**Conclusions:**

By using natural language processing of text data from image order entries, we could detect injurious falls within a shorter time than that by using incident reports. Concomitant use of this method might improve the shortcomings of an incident reporting system such as under-reporting or non-reporting and delayed submission of data on incidents.

## Background

There are various methods for identifying adverse events and patient safety incidents that have occurred in a hospital. The most reliable methodology to identify adverse events is a retrospective chart review [1]. This method provides the richest source of information on the largest number of adverse events compared to other data sources. Although this method has been widely used in epidemiological studies on adverse events, it is costly and time-consuming. In addition, judgment about adverse events in medical charts depends on the skills of reviewers [2]. Because of these shortcomings, chart review is not suitable for daily use and real-time detection of adverse events in a hospital. An incident reporting system is also widely used to identify adverse events in a hospital [3]. However, under-reporting or non-reporting is an inevitable problem in this method because the method relies on voluntary willingness of individuals [4,5]. In addition, significant lag time between adverse events and submission of incident reports impairs quick detection of adverse events [6].

Inpatient falls are the most common type of adverse events in a hospital [7]. Since 3-10% of falls in a hospital result in physical injuries of the patients including bone fractures and intracranial hemorrhage [8,9], quick identification of injurious falls is necessary. Neither chart review nor an incident reporting system is suitable for this purpose for the above-mentioned reasons. The advent of computerized physician order entry and electronic medical records has given rise to the possibility of new tools for detecting adverse events in a timely and cost-effective way [10]. Detection of adverse events such as inpatient falls from electronic medical information might resolve the shortcomings of previous methods. Since symptoms, physical findings and clinical responses are recorded as narrative text, a technique that converts narrative text data into coded form is necessary for subsequent computer-based analysis. Such a method is called natural language processing (NLP) or text mining. However, there have been only a few preliminary studies on the application of NLP to detect inpatient falls [10-12]. In those studies, adverse events such as inpatient falls were detected not by simple text searching but by an NLP algorithm from radiological reports and discharge summaries. However, there are no reports about what data source is the most suitable for detection of inpatient falls by using the NLP method.

The primary aim of this study was to determine whether it is possible to promptly detect severe injuries after falls by using the NLP method. We tried to detect fall events by using the NLP method and compared promptness of data submission and degree of injury by the events between cases recorded in incident reports and cases found by the NLP method. The second aim of this study was to determine which data source is the most suitable for the NLP method. We analyzed how many events were recorded in each data source including progress notes, order forms of diagnostic imaging and discharge summaries and how effectively the NLP method can detect fall events.

## Methods

### Settings

This study was conducted at Niigata University Hospital, an 810-bed academic hospital in the city of Niigata. There are 23 clinical departments and the service area of the hospital as a tertiary care hospital covers all districts in Niigata Prefecture, which has a population of 2,400,000.

### General overview from event occurrence to data submission

All medical, administrative and financial information of a hospital is stored and managed by using a hospital information system (HIS). The HIS is an integrated information system that is composed of backbone systems and many peripheral systems. Electronic medical records (EMRs) and physician order entries are the central components of the backbone system. The peripheral systems are specialty-specific extensions such as an incident reporting system. When a patient has fallen in the hospital, the physician who is responsible for the patient is informed of the event by medical staff, and the event is recorded in progress notes of EMRs. If the physician finds signs or symptoms that suggest injuries such as bone fracture or intracranial hemorrhage in the patient, the physician orders an x-ray examination or computed tomography scan through image order entries. On the other hand, medical staff who find falls are encouraged to report the event by using an online intra-institutional incident reporting system. Patient safety incidents and adverse events in the hospital are recorded in this reporting system. When the patient is discharged from hospital, the physician has to make a discharge summary as soon as possible. It is not mandatory to record incidents and adverse events that occurred during admission in the discharge summaries. All of the medical records and incident reports were basically written in Japanese.

### Data collection and ethical consideration

We used free-text data obtained from incident reports and from image order entries, progress notes and discharge summaries written in EMRs. Incident reports contained information on degree of injury, type of event and essential information on the event such as the name of the patient involved in the event, the name of the medical staff involved, the exact time and place, detailed description of the course of the incident, action taken by medical staff and outcome of the event. It is easy to differentiate the incident reports into fall-related and fall-unrelated events according to information on category of reports. The image order entry is a specific order form by which a diagnostic imaging test is ordered through the HIS. It contains information on possible diagnosis, short clinical course and purpose of image order. All data were obtained from a data warehouse of the HIS and were analyzed anonymously. The Ethics Committee of Niigata University School of Medicine gave ethical approval for this study.

### NLP of free-text data and construction of syntactic category rules

The free-text data were separated into sentences and analyzed by morphological analysis, which is a process of segmenting a sentence into a row of morphemes and part-of-speech (POS) tagging to each morpheme. Tagged POS data were then subjected to syntactic analysis, which is a process to determine grammatical structure with respect to given formal rules of Japanese grammar. We used grammar-driven dependency parsing to construct decision rules for distinguishing between fall-related and fall-unrelated incident reports. Each decision rule is a set of morphemes and a relationship between the morphemes. It is decided to be fall-related when a set of morphemes is detected more significantly from fall-related text than from fall-unrelated text. A comparison of the proportions of texts that fulfilled the rules among all texts between fall-related and fall-unrelated incident reports was performed by using the chi-square test and Fisher’s exact test, and the sets with significant difference (p<0.05) were selected as the decision rules. To take an example of decision rules, a decision rule (“sasaeru” + “nai” -> “tentoh”) consists of three morphemes. In this rule, “sasaeru” means “support someone” in English, “nai” means a negative signal, and “+” means that “sasaeru” and “nai” are in a dependency relation. The term “tentoh” means falling. The sign “->” indicates a cause-and-effect relationship between two events or a temporal antero-posterior relationship in the context. In summary, this rule can detect situations such as a situation in which someone cannot support a staggering patient and the patient consequently falls down. Another example is a decision rule (“bed” -> “zuri” + “ochiru”) that consists of three morphemes. In this rule, “bed” means a patient bed, “zuri” means slipping from something like a bed, and “ochiru” means falling onto the floor. Therefore, this rule can detect a situation in which a patient lying on the bed slipped from the bed and fell onto the floor. We used incident reports submitted from April 2008 to September 2008 as a training dataset to construct the decision rules. Data handling and analysis of free-text data were performed using the software Text Mining Studio Version 3.2 (Mathematical Systems Inc., Tokyo, Japan).

### Validation of the performance of category decision rules

Fall-related incident reports and fall-unrelated incident reports submitted from October 2008 to March 2009 were used as a testing dataset to validate the performance of category decision rules. The category decision rules were applied to the text data obtained from the incident reports, and sensitivity (recall), positive predictive value (PPV, precision), specificity and F-measure were calculated.

### Various data sources for detecting falls

We analyzed which type of medical records including image order entries, progress notes, discharge summaries, and incident reports are the most suitable for detecting inpatient falls when using the NLP method from two aspects. One is how many fall events were recorded in each type of medical record. The other is how effectively the category decision rules can detect fall events. Reference data of fall events (gold standard) that occurred in our hospital were obtained by a retrospective chart review by reading the above-mentioned medical records of patients who were admitted in August 2010. In order to avoid confusion of judgment, one reviewer checked all of the medical records. The reviewer is a physician and has enough experience and capability in medical record audits and adverse event analysis, because he has had a career in the Department of Medical Information and in the Department of Patient Safety. The number of patient-days in our hospital during that period was 22,401 (number of patients admitted during the period being 1,204). We analyzed how many fall events were recorded in each type of data source and calculated the proportion to the total number of true falls (data-containing rate). Next, the constructed category decision rules were applied to these text data, and data that matched the rules were selected as possible fall events. Sensitivity, PPV and F-measure were calculated.

### Comparison of lag time and degree of injury

We compared the lag time from the fall event to submission of the event to the HIS and degree of injury caused by falls. For this purpose, we used data on fall events that occurred between April 2009 and March 2010. Fall events were detected from incident reports and image order entries by using the NLP method. The falls detected by using the NLP method were confirmed by checking corresponding medical charts. The number of patient-days in our hospital during that period was 267,301 (number of patients admitted during the period being 14,448) and the number of incident reports was 4,570. Degree of injury was classified into none, mild, moderate and severe according to the conceptual framework for the international classification for patient safety [13]. Distributions of lag time were shown in medians (25-th percentile, 75-th percentile) and were compared by using Wilcoxon’s rank sum test. Proportions of falls with each degree of injury were compared by the chi-square test and Fisher’s exact test. A p-value less than 0.05 was considered significant. All statistical analyses were performed using IBM SPSS Statistics (IBM Japan Inc., Tokyo, Japan).

## Results

### Category decision rules for detection of falls

During the period from April 2008 to September 2008, 2,590 incident reports were submitted via the incident reporting system. The number of fall-related reports was 277 and the number of fall-unrelated reports was 2,313. We constructed category decision rules to distinguish fall-related and fall-unrelated reports by using these incident reports. One hundred and seventy rules were obtained, and they consisted of four major groups from syntactic views. They were rules related to fall motion (121), injuries suffered by falls (23), losing balance of the body (14) and use of fall-detecting sensors for patients at risk for falls (12).

### Performance of category decision rules

Two hundred and fifty-nine fall-related incident reports and 2,231 fall-unrelated incident reports were submitted during the period from October 2008 to March 2009. The categorical decision rules were applied to text-data obtained from these incident reports. Two hundred and twenty-four (86.5%) of the 259 fall-related incident reports were judged as containing information on fall events by the category decision rules, and 52 (2.3%) of the 2,236 fall-unrelated incident reports were considered as fall related. Therefore, sensitivity, PPV, specificity and F-measure were 0.87, 0.81, 0.98 and 0.84, respectively.

### Fall events detected from each data source

**Figure 1:**
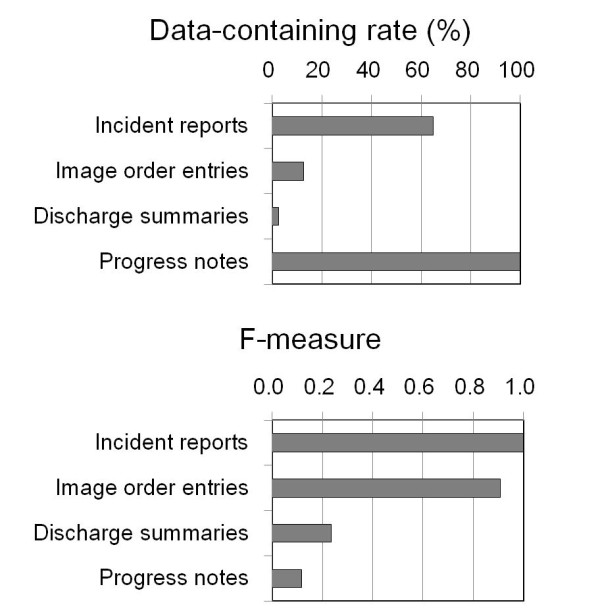
**Sensitivity and F-measure to detect falls from various sources of information.** Data-containing rate and F-measure to detect falls were compared between various sources of information in electronic medical records.

A chart review was performed for medical records of 22,401 patients (patient-days) admitted to our hospital in August 2010 (number of patients admitted during the period being 1,204). During that period, a total of 80 fall events were recorded in various types of medical records. All 80 events were recorded in progress notes, whereas 52 of these events were reported in incident reports (data-containing rate of 65.0%). Ten events were recorded in order forms of image order entries (data-containing rate of 12.5%) and only two events were recorded in discharge summaries (data-containing rate of 2.5%). We applied the categorical decision rules to each data source, and sensitivity, PPV and F-measure to detect fall events when using each data source were compared (Figure 1, Table 1). Among these data sources, incident reports were the best in terms of sensitivity (1.00), specificity (1.00), PPV (1.00) and F-measure (1.00). The second best data source was image order entries (sensitivity=0.83, specificity=1.00, PPV=1.00, F-measure=0.91). Discharge summaries (sensitivity=1.00, specificity=0.99, PPV=0.13, F-measure=0.24) and progress notes (sensitivity=1.00, specificity=0.98, PPV=0.06, F-measure=0.12) were inferior to the former two data sources. Inpatient fall rate was calculated to be 3.57 falls/1,000 patient days from the results of the chart review and the results of NLP of progress notes. In contrast, it was calculated to be 2.32 falls/1,000 patient days from the results of NLP of incident reports.

**Table 1:** Performance of the natural language processing (NLP) method in various kinds of data sources

**Data sources**	**Results of NLP analysis**	**Fall-positive**	**Fall-negative**	**Total**	**Sensitivity (Recall)**	**PPV (Precision)**	**Specificity**	**F-measure**
	Positive	223	3,351	3,574				
Progress notes	Negative	0	209,064	209,064	1.00	0.06	0.98	0.12
	Total	223	212,415	212,638				
	Positive	52	0	52				
Incident reports	Negative	0	476	476	1.00	1.00	1.00	1.00
	Total	52	476	528				
	Positive	10	0	10				
Image order entries	Negative	2	19,672	19,674	0.83	1.00	1.00	0.91
	Total	12	19,672	19,684				
	Positive	2	13	15				
Discharge summaries	Negative	0	1,149	1,149	1.00	0.13	0.99	0.24
	Total	2	1,162	1,164				

PPV means positive predictive value.

### Comparison of incident reports and image order entries as text data sources

**Figure 2:**
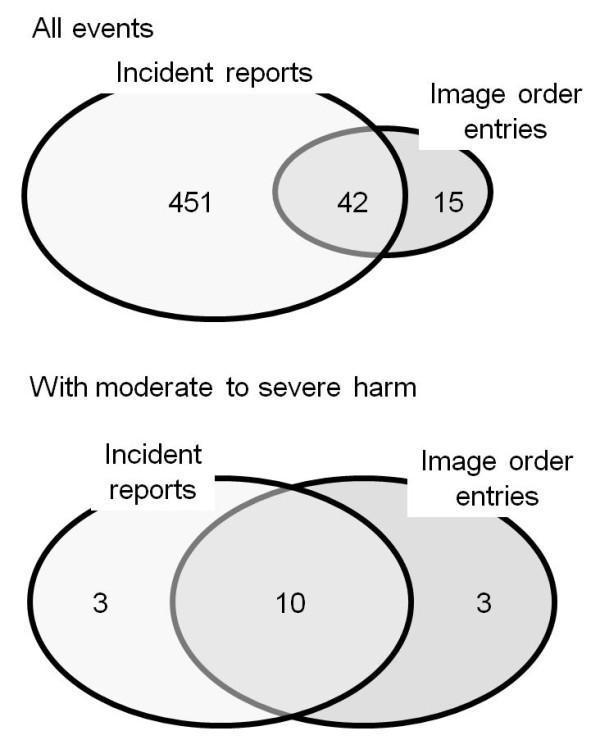
**Numbers of falls found by incident reports and NLP of image order entries.** Numbers of falls found by incident reports, image order entries and both methods are shown.

**Figure 3:**
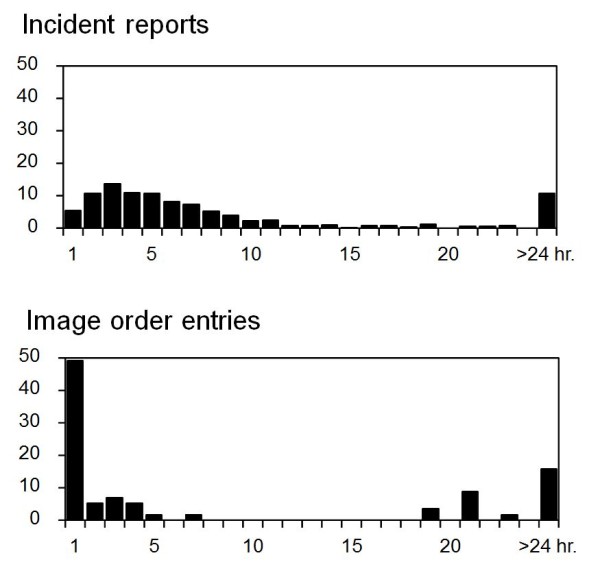
**Lag time from each fall to submission of fall data.** Distribution of lag times between falls and submission of the events to the hospital information system is shown for the incident reporting system (upper) and image order entries (lower).

**Figure 4:**
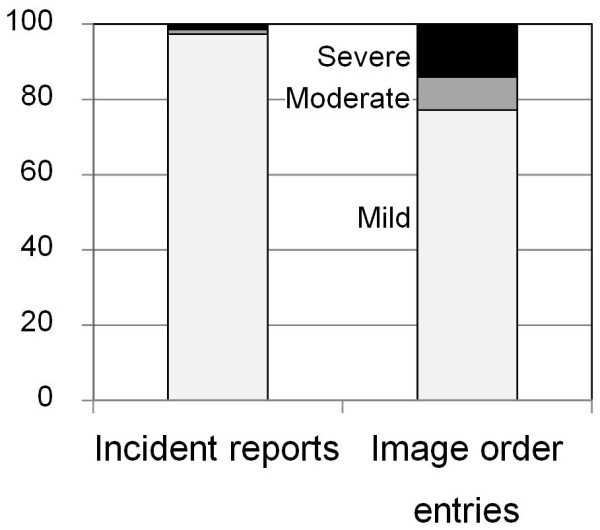
**Degrees of injury caused by falls found by the two methods.** Degrees of injury were compared between falls found from incident reports and image order entries. Proportion of falls with moderate to severe injury was significantly higher in falls found by image order entries than in falls found by the incident reporting system (p<0.001).

Since F-measure was excellent when using incident reports and image order entries as text data sources, we specifically compared the performance of NLP to detect fall events when using incident reports and image order entries as data sources. Progress notes seem unsuitable for daily use, because analysis of a large number of false-positive cases is cumbersome. Discharge summaries contain little information on fall events. We used data on 267,301 patient-days in our hospital between April 2009 and March 2010. During that period, 451 falls were detected from incident reports, 15 falls were detected from image order entries and 42 falls were detected from both data sources (Figure 2). Therefore, concomitant use of order entries and incident reports as the data source increased detection of fall events by 15 falls (3.0%) compared with that when using incident reports as the sole data source for detecting fall events. The modalities of imaging were computed tomography in 34 orders (59.6%) and x-rays in 23 orders (40.4%). Next, we compared the 493 falls detected from incident reports and the 57 falls detected from image order entries in terms of promptness to detect events and seriousness of the detected events. Median lag times between fall events and submission of the information to the HIS were 73.0 (25-percentile, 29.0; 75-percentile, 1201.0) min for image order entries and 291.0 (59.5, 510.8) min for incident reports (Figure 3). The lag time when using image order entries was significantly shorter than that when using incident reports as the data source (p<0.001). Lag time when using image order entries showed a peak at one hour and showed a second peak at 18 hours or later after falls. The second peak corresponded to cases in which symptoms from injuries became apparent after the asymptomatic period. The degrees of injury in the total of 508 events were mild in 492 events (96.9%), moderate in 7 events (1.4%) and severe in 9 events (1.8%). Among the 492 mild events, 448 (91.1%), 12 (6.5%) and 12 (2.4%) events were found by incident reports, order entries and both, respectively. The moderate cases were found from incident reports in 2 cases (28.6%), order entries in 1 case (14.3%) and both in 4 cases (57.1%). The severe cases were found from incident reports in 1 case (11.1%), order form in 6 cases (66.7%) and both in 2 cases (22.2%). Therefore, the degrees of injury in fall events found by incident reports were mild in 480 (97.4%), moderate in 6 (1.2%) and severe in 7 (1.4%) of the events. On the other hand, the degrees of injury in the 57 fall events found by order entries were mild in 44 (77.2%), moderate in 5 (8.8%) and severe in 8 (14.0%) of the events. The degree of injury in fall events detected by image order entries was significantly more severe than that in fall events detected by incident reports (p<0.001, Figure 4). Since incident reports contain many mild cases, the degree of injury might have affected the long lag time of incident reports. Therefore, we analyzed the relationship between lag time and degree of injury (Table 2). There were no differences in lag time between mild cases and moderate to severe cases both in the cases from incident reports and the cases from image order entries. On the other hand, there were significant differences in lag time between the cases found from incident reports and the cases from image order entries both in mild cases and moderate to severe cases. Therefore, the degree of injury had no influence on lag time.

**Table 2:** Comparison of lag times between incident reports and image order entries

	**Incident repots**	**Image order entries**	**Sig.**
Mild	289.5 (158.0, 507.8)	96.0 (24.0, 1230.0)	0.003
Moderate to severe	344.0 (222.3, 3542.0)	45.0 (27.0, 183.5)	0.003
Sig.	0.170	0.576	

The comparison was performed separately in mild cases and in moderate to severe cases.

### Falls with moderate to severe injuries

A total of 16 fall events with moderate to severe injuries were detected by incident reports and free text data obtained from image order entries (Figure 2). Among those cases, three cases were detected from incident reports, three cases were detected from image order entries and ten cases were detected from both data sources. Therefore, concomitant use of free text data obtained from image order entries increased the detection rate of moderate to severe fall events by 23.1%. In the three cases that were detected from only incident reports, no information on falls but only information on injuries caused by the falls was inputted in the order forms. Moderate to severe injuries caused by falls included peripheral bone fractures (9 cases), new onset vertebral compression fractures (2 cases), intracranial hemorrhage (4 cases) and disruption of surgical wounds (1 case).

## Discussion

Our results showed that narrative text data on image order entries were the most valuable data for rapid detection of injurious falls when using the NLP method to detect falls in a hospital. Although physicians did not give image orders in mild events that they considered would not involve injuries after falls, detection rate of moderate to severe injurious falls by this method was comparable to that by incident reports. In addition, lag time between incident and submission of data was significantly shorter in this method than that of incident reports. This method is suitable for rapid detection of injurious falls.

One of the most important things for coping with inpatient falls is to find patients who have suffered physical injuries after falls as soon as possible. In reality, more than 90% of inpatient falls do not result in physical injuries, but the costs attributable to falls are highly skewed to those that resulted in physical injuries [14]. Injuries after falls include bone fracture, soft tissue injuries and hematomas. These injuries may lead to additional healthcare costs, prolonged length of stay and psychological distress for the patients. This situation might result in complaints and even litigation from families of the patients [15]. In that respect, incident reports are not sufficient because of under-submission or non-submission of reports [16,17] and delayed submission of reports [6]. To overcome these shortcomings, previous reports have suggested that more than one method should be used to detect adverse events in a hospital in addition to incident reports [18]. These methods include direct observation by medical staff [19] and real-time chart review [18]. However, these methods are time-consuming and costly. Once established, NLP of image order entries could detect adverse events in a shorter time with less cost than those methods. This method can compensate for the limitations of incident reports.

Our results also showed the possibility of application of NLP of progress notes to reduce review time and labor of a manual chart review for detecting adverse events in a hospital. When the NLP method was applied to progress notes, PPV to detect fall events was 6%. However, the sensitivity to detect fall events in this case was 100%. Therefore, this 6.0% contains all fall events that were found by a manual chart review, and there was no information on fall events in the remaining 94.0% of progress notes data. If we apply the NLP method to progress notes before the manual chart review, the amount of data that we must look through would fall by 94.0%. That is, the amount of data to be checked in the manual chart review can be reduced from 100% of data to 6% of the data compared with performing a manual chart review of the original progress notes. Use of NLP of progress notes before the manual chart review might decrease labor, time and cost expended on the manual chart review without loss of information on fall events. Since our results showed that about two thirds of fall events were not reported by the incident reporting system, this method can overcome the underreporting problem of incident reports [18].

There have been reports from English-speaking countries about the application of NLP for detecting adverse events in EMRs [11]. However, there have been few such reports from non-English speaking countries including Japan [20]. To the best of our knowledge, this report is probably the first report on detection of adverse events from EMRs written in Japanese using NLP. Performance of NLP of EMRs and detection of adverse events from EMRs depend on the language the EMRs are written in. Our results showed that the method is a promising method for detecting adverse events from EMRs written in a non-English language.

Machine learning methods such as artificial neural network, support vector machine and Bayesian method have been used for classification of incident reports [21]. In these methods, algorithms for the classification are developed empirically by recognition of complex characteristics or patterns of training data through probability distributions of the data. The performance of these methods is affected to some degree by the category of incident reports, but these classifiers perform basically well in categorizing incident reports. Our approach is quite different from these methods in that it tries to detect sets of morphemes and interdependent relationships between the morphemes, which eventually aims at deep semantic analyses of text. Although our method might be a beginning to the goal, the performance of our method to categorize incident reports into fall-related and fall-unrelated was comparable to that of machine learning [21]. Further study is needed to improve the performance of this method and to widen the application of this method to other categories of adverse events.

## Conclusions

By using natural language processing of text data of image order entries, we could detect injurious falls within a shorter time than that by using the incident reporting system. Concomitant use of this method might improve the shortcomings of an incident reporting system such as under-reporting or non-reporting and delay of reporting, especially for falls with severe physical injuries.

## Competing interests

The authors declare that they have no competing interests.

## Authors’ contributions

ST is solely responsible for this manuscript.

## Pre-publication history

The pre-publication history for this paper can be accessed here:

http://www.biomedcentral.com/1472-6963/12/448/prepub
